# 

**Published:** 1893-06-17

**Authors:** 


CONTENTS.

**The Morrell Mackenzie Controversy** - 177

**Foods for Arctic Explorers** - 178

**Medical History from the Earliest Times.** By E. T. WITHINGTON, M.A., M.B., Oxon. LVIII.—The Clinicians (*continued*). - 179

**Contemporary Theory and Research** - 180

**Diet in Disease.** By Mrs. ERNEST HART. XXIII.—Food for the Aged - 181

**Gleanings in Science and Medicine** - 182

**Annotations.**—Medical Protection and Medical Defence—A Childish Quarrel at Oxford—New Treatments, New Dangers - 183

**Notes and News** - 184

**Editor's Letter Box.**—A Reminder for Absentees—The Disinfection of Wounds—The Opium Question - 191

**The Hospital Clinic**—NEWCASTLE-ON-TYNE THROAT AND EAR HOSPITAL.—Treatment of Otorrhœa (*continued*) - 185

BATH EYE INFIRMARY.—The Treatment of Cosmetic Defects.—I. 186

ROYAL FREDERICK HOSPITAL, COPENHAGEN.—The Treatment of Intestinal Cancer - 187

NEW APPLIANCES AND THINGS MEDICAL.—, "Izal"—Dr. Farr's Camphoretted Eucalyptus Medallions—Ventilating Socks - 188

**The Institutional Workshop**—

HOSPITAL SUNDAY.—What some of the Preachers said - 189

PRACTICAL DEPARTMENTS. — Warren's Cooking Pot — The "Gourmet" Boiler - 190

**Scraps and Gleanings** - 192

**The Book World of Medicine and Science** - vii

**Medical Vacancies and Appointments** - ix

EXTRA SUPPLEMENT.—THE NURSING MIRROR.

**News from the Nursing World.**—The Royal Wedding—The Nurse Dolls—University College Hospital—Interesting Cases—Sick Room Visiting—She had Come to Stay—Probationers at Bristol—Busy Bees at Barrow—Rest for Queen's Nurses—Wales—Liberality in Norway—Ambulance Service in Italy—Europeans in India—Short Items - cxi

**Sanitation for Nurses.** By A SANITARY INSPECTOR. X.—Some Recent Sanitary Provisions (*continued*) - cxiii

**Wants and Workers** - cxiii

**Nursing in India.** By OUR INDIAN CORRESPONDENT.—The Indian Nursing Service - cxiv

**London Skin Hospital, Fitzroy Square** - cxv

**Queen Victoria Jubilee Institute for Nurses.**—Rural Branch - cxiv

**Minor Appointments—Appointments** - cxv

**Presentation to Mrs. Garrett-Anderson** - cxv

**Medico-Psychological Association** - cxvi

**On the Changing of Probationers.** By A WARD SISTER - cxvi

**Trained Nurses in Workhouses** - cxvii

**Notes and Queries** - cxvii

**For Reading to the Sick.**—Suffering which leads to Glory - cxvii

**Where to Go** - cxvii

**The Muses' Looking Glass** - cxviii

**The Book World for Women and Nurses** - cxix

